# The mechanical characteristics of an aluminum foam winding CFRP composite structure under axial compression

**DOI:** 10.1016/j.heliyon.2024.e31658

**Published:** 2024-05-22

**Authors:** Hui Zhou, Yao Jiang, Guanghui Yang, Suchao Xie

**Affiliations:** a School of Logistics, Central South University of Forestry and Technology, Changsha, 410004, China; b School of Traffic & Transportation Engineering, Central South University, Changsha, 410075, China; c Key Laboratory of Traffic Safety on Track, Ministry of Education, Central South University, Changsha, 410075, China

**Keywords:** Carbon fiber reinforced plastics, Aluminium foam, Energy absorbing structures, Finite element method (FEM)

## Abstract

To enhance the energy absorption properties of the energy-absorbing structure, carbon fiber-reinforced polymer (CFRPs) with higher specific energy absorption and porous material aluminum foam with better compressive characteristics were organically combined, and a lighter aluminum foam winding carbon fiber-reinforced polymer structure (CFRP-FA-FW) was designed. Through quasi-static compression testing, the deformation mode and energy absorption properties of CFRP-FA-FW under axial load were examined. The energy absorption and specific energy absorption of CFRP-FA-FW are both increased by 113.55 % and 60.73 %, respectively, compared to the simple composite structure CFRP-FA. Finite element simulation was used for the parametric analysis of the CFRP-FA-FW structure to assess the effects of the relative density of the aluminum foam, the fiber lay-up angle, and the thickness. The results reveal that the change in the relative density of aluminum foam has little impact on the failure deformation mode of CFRP-FA-FW under axial load; the structure has a higher energy absorption capacity and a smoother energy absorption process when the fiber lay-up angle is [0°/90°]_ns_ and [45°]_ns_; the energy absorption capacity of CFRP-FA-FW is significantly improved by increasing the thickness of the carbon fiber lay-up, and the procedure is also more efficient.

**Table:** Notation list

Nomenclature	Meaning
*P*	The size of the load on the specimen (kN)
$\delta_{t}$	Effective compression distance (mm)
*m*	The mass of specimen (kg)
EA	The total energy absorbed by the specimen(kJ)
*PCF*	The value of the first peak in the load-displacement curve(kN)
*MCF*	The ratio of the total energy absorbed(kN)
*CFE*	The ratio of the average load to the initial peak crush load(Kn)
*ULC*	Undulation of load-carrying capacity
$R_{∥}^{t}$	The fiber failure parallel tensile strength
$R_{∥}^{c}$	The fiber failure parallel compressive strength
$\theta_{fp}$	The fracture angle
$R_{⊥}^{t},R_{⊥}^{c}$	The transverse tensile and compressive strength
$R_{⊥⊥},R_{⊥∥}$	The transverse and longitudinal shear strength
$p_{⊥∥}^{t,c},p_{⊥⊥}^{t,c}$	The experimental fitting parameters of the angle-of-fracture inclination
$t_{n},t_{s},\text{and}\;t_{t}$	The normal stress and two shear stresses
$E_{nn},E_{ss},\text{and}\;E_{tt}$	The normal stiffness and two shear stiffnesses
$\varepsilon_{n},\varepsilon_{s},\text{and}\;\varepsilon_{t}$	The normal strain and two tangential strains
$\left\langle t_{n}\right\rangle$	The secondary nominal stress
$t_{n}^{0},t_{s}^{0},\text{and}\;t_{t}^{0}$	The normal strength and two tangential strengths
$G_{n}^{c},G_{s}^{c},\text{and}\;G_{t}^{c}$	The critical fracture energies required to initiate delamination damage in the normal direction and the two shear directions
η	The coefficient of viscosity

## 1 Introduction

Reducing the mass of the vehicle body is one of the major ways to reduce fuel consumption, however just lowering the body mass will result in a decline in crashworthiness performance. The secret to structural design is determining how to make the vehicle body lighter while guaranteeing its crashworthiness [[1], [2], [3], [4]].

Due to their high specific strength, specific stiffness, and low material density, composite materials are frequently employed in industrial manufacturing [[5], [6], [7]]. Among them, the study of FRP has been a hot topic in the field of composites research [[8], [9], [10]]. And carbon fiber composites are always the focus of FRP research, carbon fiber-reinforced polymer (CFRPs) have a density that is only approximately one-fifth that of typical mild steel materials while having a substantially higher specific strength than most metals. Consequently, there is much potential for CFRP to be used in vehicle lightweight-design and crash safety.

Scholars have undertaken much research into the mechanical properties of CFRP. Mamalis et al. [[11], [12], [13]] investigated the collapse behavior of CFRP tubes through experiment and simulation, and the results showed that the fiber lay-up angle had a significant effect on the collapse behavior of CFRP tubes, and the CFRP tubes with lay-up angle of [±0°]_ns_ and [±90°]_ns_ showed better energy absorption properties under load. Kim et al. [14] studied the energy absorption effects of carbon fiber, Kevlar fiber, and carbon-Kevlar fiber hybrid composite tubes under crushing load and found that carbon fiber tubes had the best energy absorption characteristics. Sharma et al. [15] studied the effect of separation of fiber orientation through the thickness of thin composites on their low velocity impact, and found that the [0/90/90/0] composite is having a comparatively more lateral spread of delamination and inter-layer opening than that of [90/-45/45/0] composite. Wang et al. [16] examined the failure behavior of thin-walled beam structures made of glass fiber composites under quasi-static and impact loads, and it was found that the average crush force of the thin-walled beam structures under impact loads was lower than that of the structures under quasi-static loads. Russo et al. [17] proved the influence of fiber and matrix breakage on the interlaminar damage evolution with Hashin Criteria and study the role of the intra-laminar damages in delamination propagation. Atthapreyangk et al. [18] investigated by simulation the energy absorption of thin-walled carbon fiber tubes with a lay-up angle set to [0°/90°]_ns_. The results showed that the specific energy absorption was maximum when the thickness-to-diameter ratio was 0.092. Liu et al. [19] investigated the impact response and residual performance of thin-walled CFRP tubes and aluminum tubes subjected to multiple axial impacts, and the results showed that CFRP tubes have better energy absorption performance compared to aluminum tubes under repetitive impacts and residual crush tests. Alia et al. [20] investigated the compressive properties and characterized energy absorption of carbon fiber-reinforced honeycomb structure prepared by vacuum-assisted resin transfer molding method. Zhu et al. [21] studied the crashworthiness and energy absorption characteristics of CFRP multicellular structure under quasi-static axial load and found that with the increase in the number of cells and the number of layers of internal beams, the absorption energy gradually increases. Ren et al. [22] designed a CFRP structure with open holes and investigated the crushing behavior of perforated CFRP tubes by inner spreading triggers with different trigger radii, and the results showed that the inner spreading triggers could realize the transition of the perforated CFRP tubes from peri-peripheral crushing to progressive crushing, which led to a more adequate energy dissipation performance of the material.

Aluminum foam is made of aluminum (or aluminum alloy) as a substrate, which consists of a composite structure containing metal and holes [23]. Due to its special internal porous structure, it has good impact resistance and can absorb a large amount of kinetic energy at the moment of impact, making it an ideal cushioning and energy-absorbing material [[24], [25], [26], [27]]. Zhu et al. [28] examined the impact resistance of circular aluminum tubes with internally reinforced composite skeleton and aluminum foam under quasi-static axial loading. The experimental results showed that the specific energy absorption of the structure was increased by 32 % compared to that of an empty tube. Durate et al. [[29], [30], [31]] investigated the energy absorption effect and mechanical response of metallic thin-walled tubes filled with aluminum foam under quasi-static loading conditions, and the results showed that the interactions between the aluminum foam and the metallic tubes resulted in the filled tubes having better mechanical properties and the ability to absorb and dissipate energy. Hangai et al. [32] used the friction stirring method to prepare the energy-absorbing structure of aluminum foam-filled thin-walled metal tubes, and the results showed that this method of preparation resulted in strong metallurgical bonding between the aluminum foam and the metal tubes, which enhanced the strength under compressive loading and made the energy-absorbing process smoother. Kim et al. [33] investigated the energy absorption properties and bending failure behavior of aluminum (Al)/CFRP short square hollow section beams under transverse quasi-static loading. Sun et al. [34] proposed four different hybrid sandwich tubes and investigated their durability and performance-to-cost ratios under quasi-static axial conditions. Ying et al. [35] experimentally investigated the axial collapse characteristics and energy absorption characteristics of second-order hybrid multicellular aluminum-filled CFRP (Al/CFRP) graded tubes under quasi-static loading, and the results showed that the multicellular tubes underwent a specific progressive folding, and the energy absorption capacity of the graded cross-section was significantly increased.

In summary, the current research on energy-absorbing structures on vehicles mainly focuses on a single fitting from material properties, component filling, simple combination of several materials, *etc*., and the research results obtained are limited to the optimal energy-absorbing properties of a single combination of fittings, which makes it difficult to bring the advantages of various materials and structures into full play [36-37], however CFRPs have a higher brittleness, which means that under impact or stress concentration, CFRPs are more likely to fracture rather than undergo plastic deformation. Foam aluminium, on the other hand, has relatively lower compressive and tensile strengths, which not be suitable for applications under high load conditions. CFRPs have good mechanical properties and toughness as well as excellent processability, which can provide specific solutions for different needs [38]. Compared with the simple superposition of metal thin-walled tube, aluminum foam, and CFRP, to further improve the energy-absorbing efficiency, an organic combination of CFRP and porous foam material was made, and the filament winding process was adopted to design and obtain the aluminum foam winding CFRP structure (CFRP-FA-FW), its energy-absorbing properties go beyond the simple addition of these two materials. To improve the energy absorption efficiency, the failure modes and energy absorption characteristics of the traditional structure and CFRP-FA-FW structure was firstly researched through experiments, and then parametrically analysis the relative density of the foam aluminum, the angle of CFRP layups, and the thickness of the CFRP composite structure based on the finite element model. Table 1 shows an overview of comparison between the relevant literature.

**Table 1:** Comparison between the relevant literature.

Research target	Curing method	Layering sequence	Reference
CFRP tubes	Hot pressing curing	Satin Fabric	[7,[11], [12], [13],^15^]
Carbon-Kevlar fiber hybrid composite tubes	High-pressure autoclave vacuum curing	/	[14]
Glass fiber thin-walled beam	High-temperature curing	/	[16]
CFRP tubes and aluminum tubes	Hot pressing curing	Plain Weave Fabric	[19]
Carbon fiber- reinforced honeycomb	Room-temperature curing	[0/45/-45/0]	[20]
CFRP multicellular structure	High-temperature curing	/	[21]
CFRP structure with open holes	Hot pressing curing	Plain Weave Fabric	[22]
Aluminum foam winding CFRP composite structure	Vacuum-assisted high-temperature curing	[45°/-45°]ns	This paper

## 2 Specimen and test results

### 2.1 Sample preparation

**Fig. 1:**
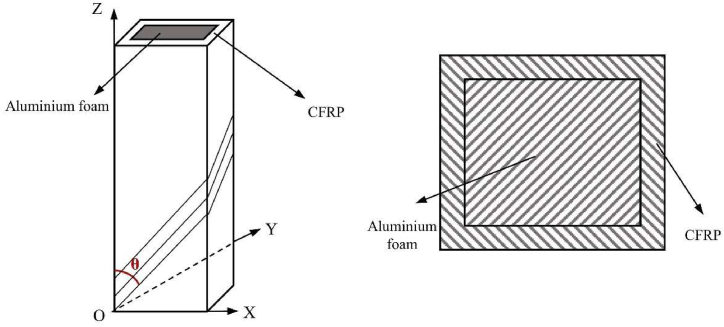
Schematic representation of the CFRP-FA-FW structure.

The CFRP-FA-FW construction is depicted schematically in Fig. 1: The CFRP was wound around the aluminum foam, and vacuum high-temperature curing was used to combine the two materials. The CFRP material has 16 layers, a single layer thickness of 0.15 mm, and a tolerance on its dimensions of less than ±0.03 mm. The angle θ between the carbon fiber and the composite structure along the axis is known as the “lay-up angle”. The CFRP-FA-FW specimen was prepared using the vacuum bagging technique, and the preparation procedure mostly entails preparing the molds and materials, carbon fiber lay-up, sealing the made-up part, injecting resin, vacuum high-temperature curing, cooling, and demolding.

**Fig. 2:**
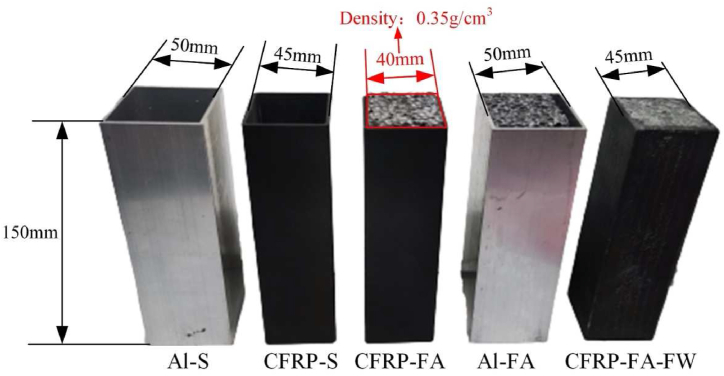
Quasi-static axial compression specimens.

Fig. 2 displays several test specimens, including an aluminum alloy square tube (Al–S), CFRP square tube (CFRP-S), CFRP square tube filled with aluminum foam (CFRP-FA), an aluminum tube filled with aluminum foam (Al-FA), and a T300-grade CFRP-FA-FW, in which the side length of an aluminum alloy square tube is 50 mm by 50 mm, and the thickness is 2 mm; the side length of a carbon fiber tube is 45 mm by 45 mm, and the thickness is 2.5 mm; the density of an aluminum foam is 0.35 g/cm^3^, and the side length is 40 mm by 40 mm, and the total length of all specimens is 150 mm.

### 2.2 Indices for evaluating energy absorption properties

This paper primarily introduces the following evaluation indices: total energy absorption (*EA*), specific energy absorption (*SEA*), initial peak crush force (*PCF*), mean crush force (*MCF*), compression force efficiency (*CFE*), and undulation of load-carrying capacity (*ULC*). These indices are used to compare the energy absorption characteristics of various structures under axial loads.

(1) Total energy absorption (*EA)*: the total energy absorbed by the specimen within the effective compression distance, EA can be calculated using Eq. (1):

(1) $$EA=\int_{0}^{\delta_{t}}Pd\delta$$

where: *P* denotes the magnitude of the load on the specimen at a certain moment in the compression process; δ represents the displacement at that moment; $\delta_{t}$ is the effective compression distance at the end of the specimen compression.

(2) Specific energy absorption (*SEA*): the ratio of the total energy absorbed by its mass, which is given by Eq. (2):

(2) $$SEA=\frac{EA}{m}$$

where: *m* is the mass.

(3) Initial *PCF*: the value of the first peak in the load-displacement curve during the compression process.
(4) *MCF*: the ratio of the total energy absorbed by the effective compression distance, which can be calculated using Eq. (3):

(3) $$MCF=\frac{EA}{\delta_{t}}$$

(5) *CFE*: the ratio of the average load to the initial peak crush load, which can be used to evaluate the consistency of the load during compression, which is given by Eq. (4):

(4) $$CFE=\frac{MCF}{PCF}\times 100\%$$

(6) Undulation of load-carrying capacity (*ULC*): the degree of undulation of the instantaneous load relative to the average load, ULC can be calculated using Eq. (5):

(5) $$ULC=\frac{\int_{0}^{\delta_{t}}\left|P-MCF\right|d\delta }{EA}$$

### 2.3 Quasi-static axial compression test results

**Fig. 3:**
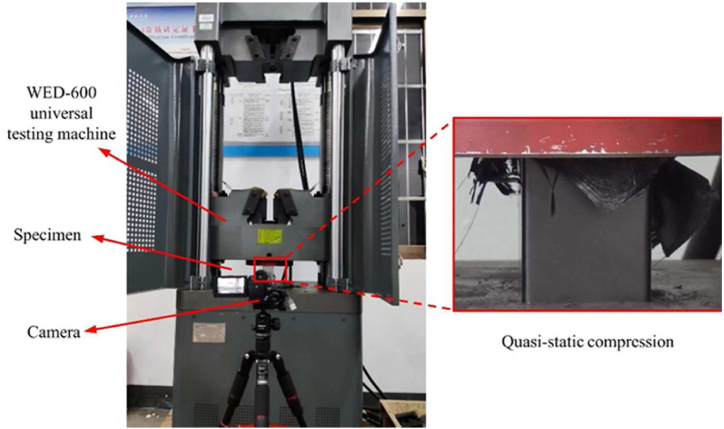
Quasi-static axial compression test set-up.

This test was conducted on a WED-600 electro-hydraulic servo-motor-controlled universal testing machine (Fig. 3), at a cross-head displacement rate of 6 mm/min.

**Fig. 4:**
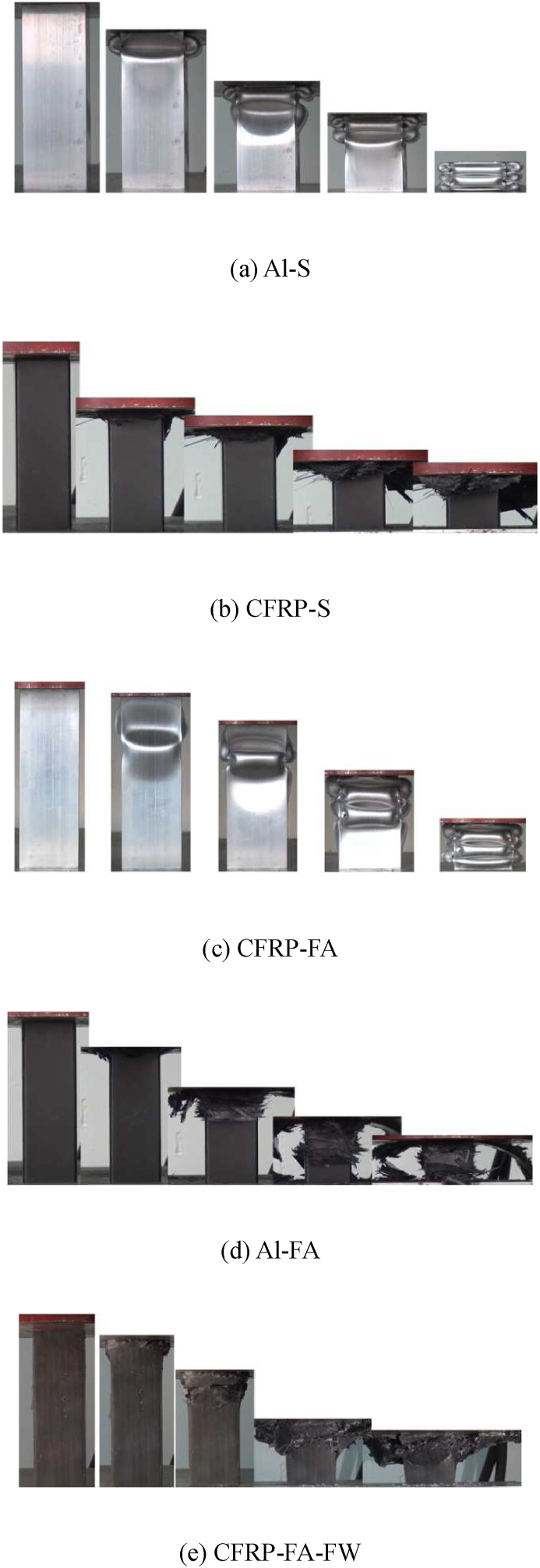
Axial compression deformation of each specimen.

Fig. 4 depicts the axial compression-induced deformation of each specimen. According to Fig. 4(a), the aluminum alloy square tube exhibits uniform fold deformation from the end, and after exhibiting three periodic folds, the compression collapse of the aluminum alloy square tube reaches the densification stage. CFRP provides energy dissipation primarily through fiber failure, resin matrix failure, and complicated laminations. Fig. 4(b) depicts the axial compression collapse deformation diagram for the [45°/-45°]_ns_ lay-up angle carbon fiber square tube. According to the deformation diagram, the end brittle fractures of carbon fiber square tubes with the movement of the rigid plane, lead to carbon fiber fragments that are coiled both inside and outwards. The carbon fiber square tube deforms in a progressive compression collapse mode. The four corners of the square tube split apart to absorb energy, and the entire carbon fiber square tube splits into four blades that stretch and bend downward. Each piece of carbon fiber exhibits clear delamination.

As seen in Fig. 4(c), which depicts the axial collapse deformation of the aluminum tube filled with aluminum foam, the overall structure still displays an accordion-type asymmetric deformation pattern under axial stress, resulting in orderly fold deformation. The folds of the filled structure are more compact than those of a single aluminum alloy square tube because the aluminum foam restrains the inward deformation of the aluminum tube.

Fig. 4(d) depicts the axial collapse deformation of a carbon fiber square tube filled with aluminum foam. The failure deformation of the filler structure is akin to that of a single carbon fiber square tube overall, and it is characterized by a “blossoming” type of failure with splitting in the four corners of the carbon fibers and delamination of the blades on each side wall. The inwardly curved blades, however, cannot be extended due to the structure being filled with aluminum foam, and the degree of fragmentation exceeded that of the single carbon fiber tube, resulting in the production of finer debris.

The axial compression deformation of the CFRP-FA-FW structure is depicted in Fig. 4(e). The carbon fibers in the outer layer did not separate and peel off as in Fig. 4(d) but instead created a stacked-shrinkage deformation resembling that of the aluminum alloy square tube because of the closer connection between the carbon fibers and the aluminum foam. This is primarily because, during the curing process of composite structures, some resins are also injected between the carbon fiber layer and the aluminum foam, creating a stronger link between the two materials. In addition, the aluminum foam has numerous holes on its surface, and the filament winding process increases the contact area between the carbon fibers and the aluminum foam. As a result, in addition to the crushing deformation of the carbon fibers themselves and the plastic deformation of the aluminum foam, as well as the failure of the resin between the carbon fibers and the aluminum foam, all three forms are involved in the energy dissipation together to increase the energy-absorbing effect.

**Fig. 5:**
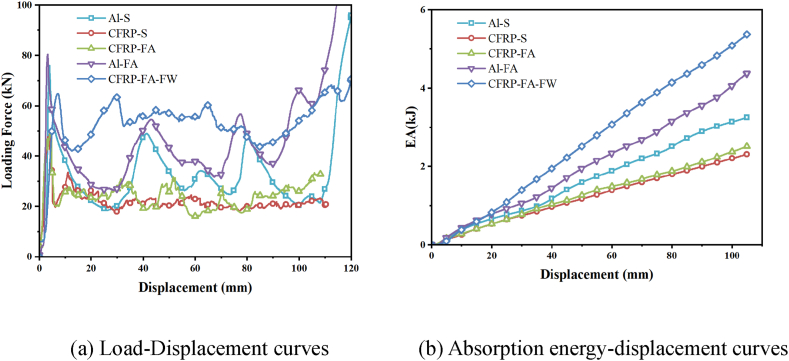
Comparison of compression results of different structures.

The comparison of the axial crush load-displacement curves of various structures is shown in Fig. 5(a). While the undulation of the load-carrying capacity of an aluminum alloy structure is larger, the CFRP-FA-FW absorbs energy more smoothly and exhibits lower-amplitude undulations. This is primarily because the two materials’ energy-absorption mechanisms differ. While aluminum alloy material dissipates energy by producing pleats that transform kinetic energy into plastic deformation energy, CFRP primarily absorbs energy through modes such as splitting, which microscopically manifests as a complex compression-crushing process such as fracture of carbon fibers, detachment of fibers from the substrate, and delamination of plies. The energy absorption-displacement curves for various structures are shown in Fig. 5(b) under axial loads. As can be observed, the CFRP-FA-FW construction absorbs significantly more energy than the other four structures.

**Fig. 6:**
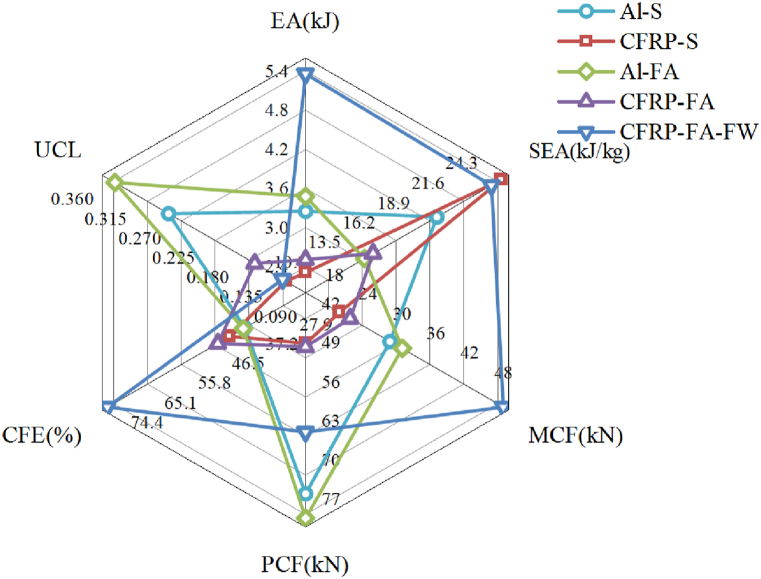
Radar chart for comparison of indicators.

Comparing the evaluation indices of the energy-absorption properties of five different structures (Table 2 and Fig. 6), the CFRP-FA-FW structure shows a higher *MCF* and a lower initial *PCF* than the Al–S structure and the Al-FA structure in the axial loading process. Additionally, the *PCF* and *MCF* of the CFRP-FA-FW structure have improved differences of 65.01 % and 54.29 %, respectively. In comparison to Al–S and Al-FA, the *MCF* of the CFRP-FA-FW is 65.01 % and 54.29 % higher, respectively. Additionally smoother in terms of energy absorption, the CFRP-FA-FW has a *CFE* that is significantly greater than that of the other constructions at 78.69 %. The *ULC* of the CFRP-FA-FW structure is 0.12, which is just slightly higher than that of the CFRP-S structure, which also absorbs energy more smoothly. In comparison to CFRP-S, which also has a smoother energy absorption process, and to CFRP-FA, which is also a filler structure composed of a straightforward mix of carbon fibers and aluminum foam, the *ULC* of the CFRP-FA-FW structure is 0.12, which is only up 0.01 and down 0.05, respectively. Regarding the energy absorption indices, the energy-absorbing structures made of carbon fibers had higher specific energy absorption, with the CFRP-S and CFRP-FA-FW having values of 25.43 kJ/kg and 24.64 kJ/kg, respectively.

**Table 2:** Comparison of evaluation indices of energy absorption characteristics of different.

Structures	*m*(g)	*EA*(kJ)	*SEA*(kJ/kg)	*MCF*(kN)	*PCF*(kN)	*CFE*(%)	*ULC*
Al–S	159.77	3.25	20.34	30.95	76.00	40.72	0.27
CFRP-S	90.82	2.31	25.43	21.99	49.10	44.80	0.11
Al-FA	237.20	3.48	14.65	33.10	80.40	41.17	0.34
CFRP-FA	163.85	2.51	15.33	23.93	49.70	48.14	0.16
CFRP-FA-FW	217.64	5.36	24.64	51.07	64.90	78.69	0.12

## 3 Finite element model and validation

### 3.1 Finite element model

**Fig. 7:**
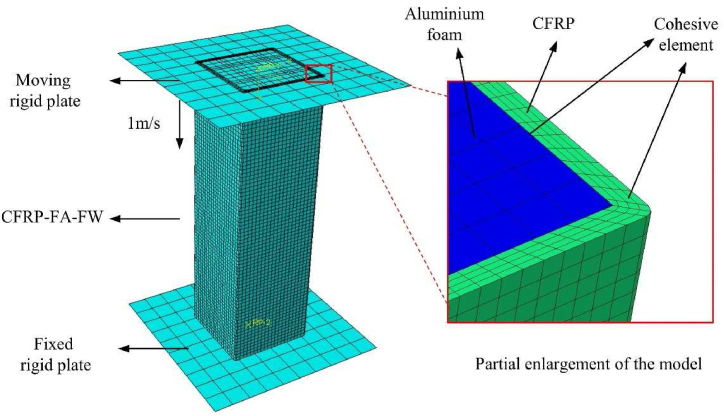
Finite element model of CFRP-FA-FW.

**Fig. 8:**
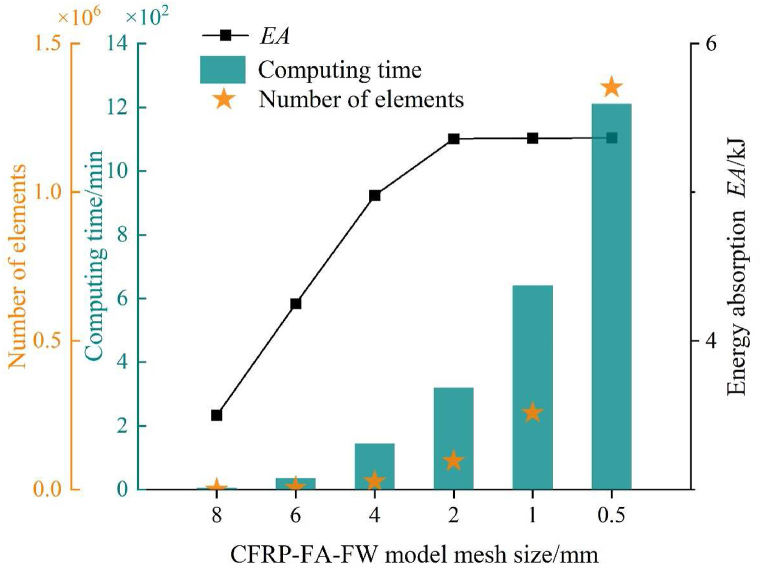
Mesh sensitive analysis of CFRP-FA-FW model.

The finite element model of the CFRP-FA-FW structure has been established (Fig. 7). The 2.5 mm carbon fiber tube is divided into five layers, each with a thickness of 0.5 mm, and the lay-up angles are alternately set at 45° and −45°. As shown in Fig. 8, through the mesh size sensitive analysis of the CFRP-FA-FW model, it is learned that the *EA* of the tube absorption energy has shown convergence when the mesh size is 2 mm. And the number of meshes and the computation time increase dramatically as the mesh size continues to decrease, so the subsequent analysis will be carried out with the model with a mesh size of 2 mm. The model is then cut according to the thickness of the carbon fiber lay-up and the lay-up settings. With a cell size of 2 mm × 2 mm, a continuous shell cell (SC8R) and a cohesion cell with zero thickness (COH3D8) are used to mimic the carbon fiber layers and interlayer forces. The aluminum foam is simulated in ABAQUS using a solid cell (C3D8R), as it was impossible to mimic the foam material based on its fine structure [39,40]. And the material model is selected as Crushable Foam. Crushable Foam with specific energy absorption properties can be designed by varying parameters such as density, porosity, pore size and distribution of the foam. The Crushable Foam model is based on the uniaxial compression and axisymmetric triaxial compression tests conducted by Deshpande on closed-cell metallic foam aluminum, and the self-similar intrinsic model of the foam material based on the von Mises yield criterion, whose intrinsic equations are shown in Eq. (6)

(6) $$f=\sqrt{\frac{\sigma_{eq}^{2}+\alpha^{2}\sigma_{m}^{2}}{1+{\left(\alpha /3\right)}^{2}}}-Y\leq 0$$

The closed-cell pore size range in aluminum foam is mainly between 2.5 mm and 5 mm, and its porosity is 84.24 %. A fixed rigid plane below and a moving rigid plane above, which can be moved downwards at 1 m/s, respectively, are used to constrain the CFRP-FA-FW structure. ABAQUS display computation, in which the display solution method is computed using the central difference method has been taken for simulation, the computation time is determined by the stabilization time increments Δt, the larger Δt is, the faster the calculation is. The expression for Δt is shown in Eq. (7):

(7) $$\Delta t=\frac{L^{e}}{c_{d}}$$

where $L^{e}$ is the length of the smallest grid cell, $c_{d}$ is the expansion wave velocity of the material, as shown in Eq. (8)

(8) $$c_{d}=\sqrt{\frac{E}{\rho }}$$

where *E* is the modulus of elasticity and ρ is the material density. Thus, scaling up the mass reduces the expansion wave velocity, which increases Δt and shortens the computation time. The kinetic energy of the system must not be greater than 5 % of the total internal energy when using the mass enlargement method to increase computing efficiency. The friction coefficient is set to 0.2 and all modelled connections are configured as universal contacts.

### 3.2 Material modelling

#### 3.2.1 CFRP model

A multilayer shell unit damage model of CFRP is created to predict the failure behavior of the material. An intralaminar damage sub-model and an interlaminar damage sub-model comprise the bulk of the model. The interlaminar damage sub-model is based on a cohesive zone model in ABAQUS to simulate the delamination phenomena of the CFRP under axial load. The intralaminar damage sub-model is used to simulate the failure modes of the fiber and resin matrix, and the calculation relies on a user-defined VUMAT subroutine based on the Puck failure criterion. Puck's failure criterion is based on the physical importance and damaging phenomenon of the two types of composite plywood failure: fiber fracture (FF) and inter-fiber fracture (IFF) [41].

The Puck failure criterion uses the maximum stress failure criterion to identify the damage state of fibers and define the failure state. When the stress parallel to the direction of the fiber is equal to or higher than the stress necessary for the fiber to fail under the action of uniaxial stress loading, the fiber fails under the influence of multi-directional stress loading, according to this failure criterion and its implied physical meaning. This presumption leads to the following equation for the fiber failure criterion:

(1) When $\sigma_{1}＞0$, the fiber is stretched, $f_{E,FF}$ needs to satisfy Eq. (9):

(9) $$f_{E,FF}=\frac{\sigma_{1}}{R_{∥}^{t}}\geq 1$$

(2) When $\sigma_{1}＜0$, the fiber is in compression, $f_{E,FF}$ needs to satisfy Eq. (10):

(10) $$f_{E,FF}=\frac{\sigma_{1}}{-R_{∥}^{c}}\geq 1$$

where $R_{∥}^{t}$ is the fiber failure parallel tensile strength and $R_{∥}^{c}$ is the fiber failure parallel compressive strength.

**Fig. 9:**
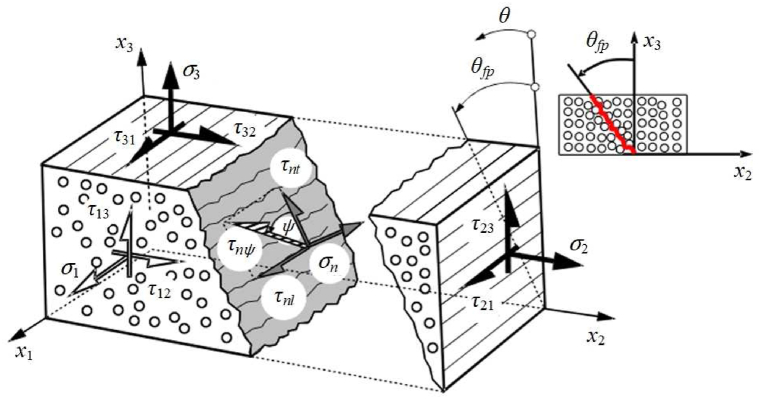
The stress and fracture angle at the acting surface.

The Puck failure criterion employs a coordinate system based on the fracture plane of the composite plywood the more accurately to define the inter-fiber failure state (Fig. 9). The laminate coordinate system is rotated θ by around the $x_{1}$-axis to produce this coordinate system on the acting surface. If this surface undergoes fracturing, this surface will then serve as the fracture surface, and the angle $\theta_{fp}$ will then serve as the fracture angle. The stress condition on the fracture surface is crucial for inter-fiber failure analysis since the resin in CFRP is a brittle material.

**Fig. 10:**
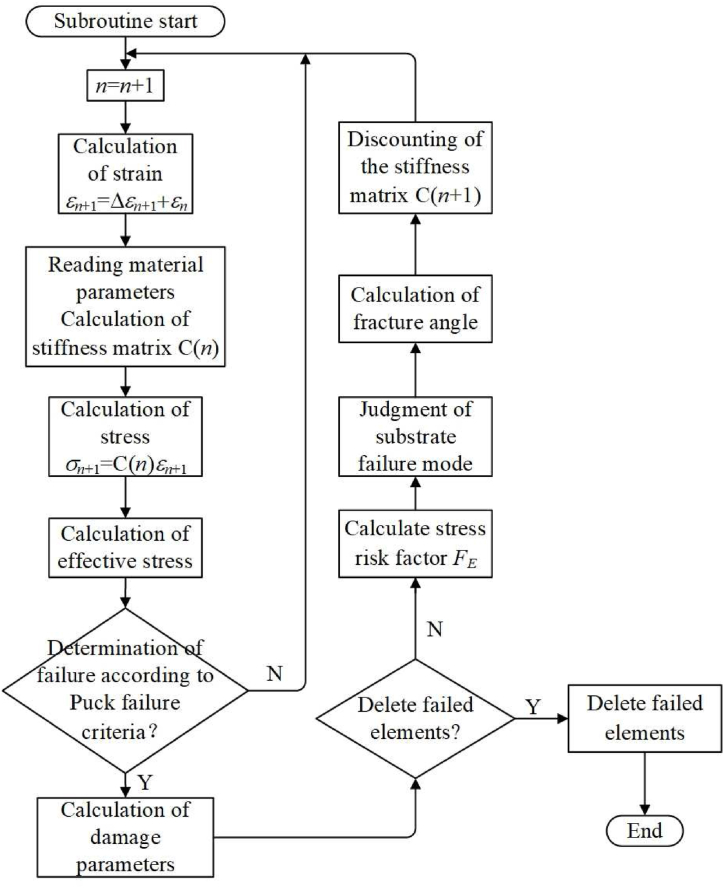
Flowchart for modelling CFRP damage and failure.

In the surface of fracture, the combined effect of the normal stress $\sigma_{n}\left(\theta \right)$ and the two shear stresses $\tau_{nt}\left(\theta \right)$ and $\tau_{nl}\left(\theta \right)$ determine the inter-fiber failure state. By using Eqs. (11), (12), (13), $\sigma_{n}\left(\theta \right)$, $\tau_{nt}\left(\theta \right)$, and $\tau_{nl}\left(\theta \right)$ can be calculated.

(11) $$\sigma_{n}\left(\theta \right)=\sigma_{2}\;\cos^{2}\;\theta +\sigma_{3}\;\sin^{2}\;\theta +2\tau_{23}\;\sin\;\theta \;\cos\;\theta$$

(12) $$\tau_{nt}\left(\theta \right)=-\sigma_{2}\;\sin\;\theta \;\cos\;\theta +\sigma_{3}\;\sin\;\theta \;\cos\;\theta +\tau_{23}\left(\cos^{2}\;\theta -\sin^{2}\;\theta \right)$$

(13) $$\tau_{nl}\left(\theta \right)=\tau_{31}\;\sin\;\theta +\tau_{21}\;\cos\;\theta$$

The shear stresses $\tau_{nt}\left(\theta \right)$ and $\tau_{nl}\left(\theta \right)$ can be combined into a single shear stress $\tau_{n\psi }\left(\theta \right)$, described by the angle ψ. The $\tau_{n\psi }\left(\theta \right)$ is given by Eq. (14):

(14) $$\tau_{n\psi }\left(\theta \right)=\sqrt{\tau_{nt}^{2}\left(\theta \right)+\tau_{nl}^{2}\left(\theta \right)}$$

According to the relationship between the transverse stress $\sigma_{2}$ and the shear stress $\tau_{21}$, the Puck failure criterion can divide the inter-fiber failure condition into the three inter-fiber failure modes of Mode A, Mode B, and Mode C. The IFF failure criterion can be stated as follows in light of the many failure scenarios mentioned above:

(1) Mode A, the $f_{E,IFF}$ can be calculated using Eq. (15):

(15) $$f_{E,IFF}=\sqrt{{\left[\left(\frac{1}{R_{⊥}^{t}}-\frac{p_{⊥∥}^{t}}{R_{⊥∥}}\right)\cdot \sigma_{2}\right]}^{2}+{\left(\frac{\tau_{21}}{R_{⊥∥}}\right)}^{2}}+\frac{p_{⊥∥}^{t}}{R_{⊥∥}}\cdot \sigma_{2}$$

(2) Mode B, the $f_{E,IFF}$ can be calculated using Eq. (16):

(16) $$f_{E,IFF}=\sqrt{{\left(\frac{\tau_{21}}{R_{⊥∥}}\right)}^{2}+{\left(\frac{p_{⊥∥}^{c}}{R_{⊥∥}}\cdot \sigma_{2}\right)}^{2}}+\frac{p_{⊥∥}^{c}}{R_{⊥∥}}\cdot \sigma_{2}$$

(3) Mode C, the $f_{E,IFF}$ can be calculated using Eq. (17):

(17) $$f_{E,IFF}=\left[{\left(\frac{\tau_{21}}{2\left(1+p_{⊥∥}^{c}\right)R_{⊥∥}}\right)}^{2}+{\left(\frac{\sigma_{2}}{R_{⊥}^{c}}\right)}^{2}\right]\frac{R_{⊥}^{c}}{\left(-\sigma_{2}\right)}$$

The fracture angle $\theta_{fp}$ in mode C is given by Eq. (18):

(18) $$\cos\;\theta_{fp}=\sqrt{\frac{1}{2\left(1+p_{⊥⊥}^{c}\right)}\cdot \left[{\left(\frac{R_{⊥⊥}^{A}\cdot \tau_{21}}{R_{⊥∥}\cdot \sigma_{2}}\right)}^{2}+1\right]}$$

where $R_{⊥}^{t}$ is the transverse tensile strength, $R_{⊥}^{c}$ is the transverse compressive strength, $R_{⊥⊥}$ is the transverse shear strength, $R_{⊥∥}$ is the longitudinal shear strength, and $p_{⊥∥}^{t,c}$ and $p_{⊥⊥}^{t,c}$ are the experimental fitting parameters of the angle-of-fracture inclination, and the typical parameters of angle-of-fracture inclination of composite laminates are summarized in Table 3.

**Table 3:** Plywood fracture angle inclination parameters.

Type	$p_{⊥∥}^{t}$	$p_{⊥∥}^{c}$	$p_{⊥⊥}^{t}$	$p_{⊥⊥}^{c}$
CFRP/Epoxy	0.35	0.3	0.25–0.30	0.25–0.30

A damage failure model with the Puck failure criterion is established based on the above description of the CFRP failure ontology model and failure criterion. The application of the failure model is realized through the user subroutine (VUMAT) interface of ABAQUS/Explicit software, and the process of simulating the CFRP damage and failure using finite elements is illustrated in Fig. 10.

**Fig. 11:**
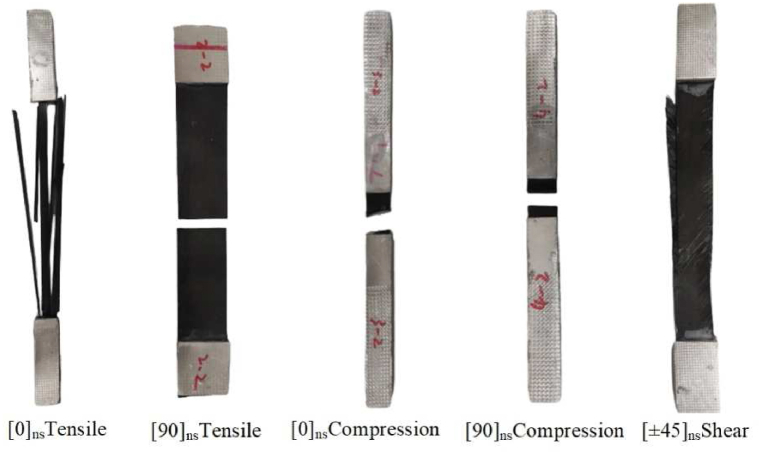
Failed specimen in tension, compression, and shear.

Unidirectional tape T300 CFRP was used to make the specimens, and epoxy resin was used to cure them. The specimens were made in accordance with ASTM D3039 [42], ASTM D6641 [43], and ASTM D3518 [44], and they were tested for the tensile, compressive, and shear properties of the carbon fiber laminate. The tests were conducted on an MTS Landmark high-frequency fatigue testing equipment, which was loaded at a cross-head displacement rate of 2 mm per minute until the specimen cracked. Fig. 11 illustrates the types of failure seen.

**Fig. 12:**
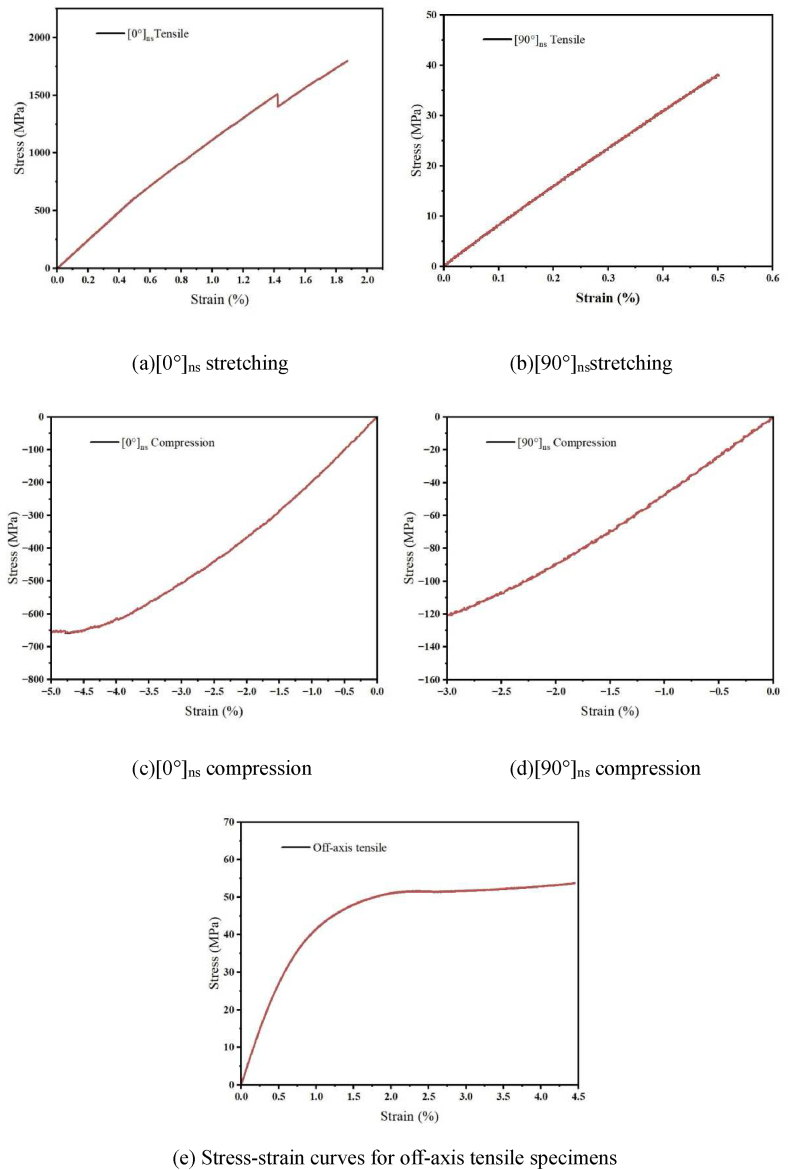
Experimental stress-strain curve of carbon fiber plywood.

Fig. 12(a–e) displays the experimental stress-strain curves for carbon fiber laminates in tension, compression, and shear.

As indicated in Table 4, the material characteristics of carbon fibers were determined using the calculation techniques described in the ASTM D3039, ASTM D6641, and ASTM D3518 standards.

**Table 4:** Material parameters of the sub-model within the layer.

Characteristics	Variable	Numerical Values
Density(kg/m^3^)	ρ	1600
Elasticity parameter(GPa)	$E_{1+}$	105.67
	$E_{1-}$	16.98
	$E_{2+}$	7.57
	$E_{2-}$	4.12
	$G_{12}$	5.24
Poisson's ratio	$\nu_{12}$	0.03
Strength(MPa)	$X_{1+}$	1504
	$X_{1-}$	34.91
	$X_{2+}$	654.08
	$X_{2-}$	126.67
	S	53.69

#### 3.2.2 Properties of aluminum foam material

**Fig. 13:**
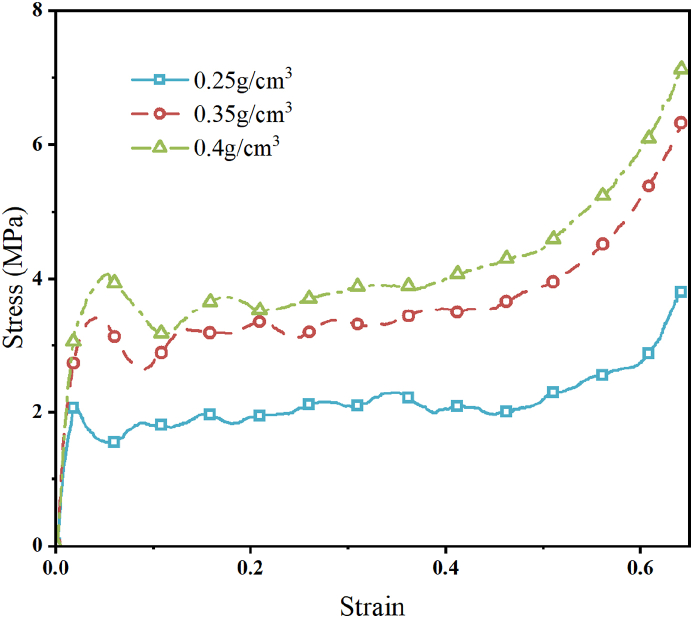
The stress-strain curve of aluminum foam.

As the main component of the CFRP-FA-FW structure in the trials, three different types of closed-cell aluminum foams with relative densities of 0.25 g/cm^3^, 0.35 g/cm^3^, and 0.4 g/cm^3^ were used in the present research. The stress-strain curves of the three types of aluminum foam were obtained (Fig. 13) as a result of the quasi-static compression experiments of the aluminum foam conducted on a universal testing machine.

#### 3.2.3 Cohesion unit models

**Fig. 14:**
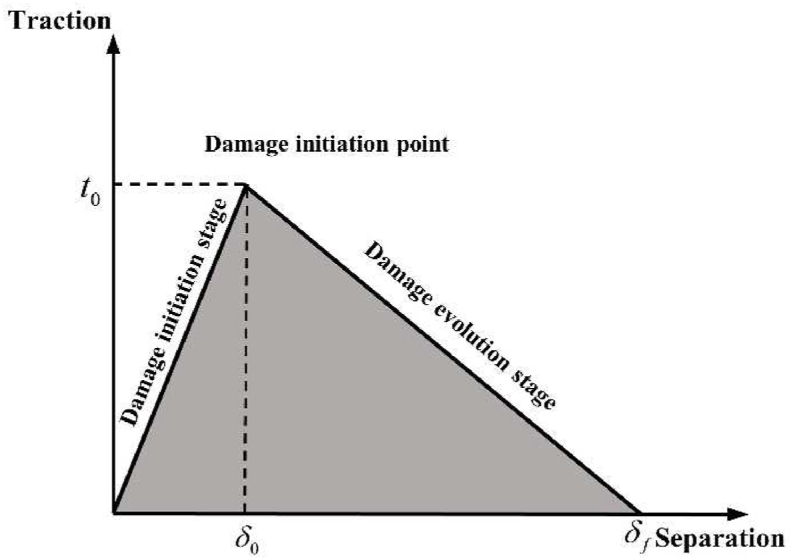
Schematic representation of the law of traction separation.

Cohesive cells (COH3D8) between fiber lay-ups are defined in ABAQUS to simulate CFRP interlaminar damage. As depicted in Fig. 14, the cohesive unit model specifies the traction separation law on the upper and bottom surfaces of the cohesive unit. The function of the cohesive unit in the simulation is divided into the damage initiation stage and the damage evolution stage. Before the damage initiation point, the intrinsic structure of the cohesive unit is linear elasticity, and Eq. (19) describes its stress-strain relationship.

(19) $$t=\left\{\begin{array}{c} t_{n}\\ t_{s}\\ t_{t} \end{array}\right\}=\left[\begin{array}{ccc} E_{nn} & 0 & 0\\ 0 & E_{ss} & 0\\ 0 & 0 & E_{tt} \end{array}\right]\left\{\begin{array}{c} \varepsilon_{n}\\ \varepsilon_{s}\\ \varepsilon_{t} \end{array}\right\}=E\varepsilon$$

where $t_{n}$ , $t_{s}$, and $t_{t}$ are the normal stress and two shear stresses; $E_{nn}$, $E_{ss}$, and $E_{tt}$ are the normal stiffness and two shear stiffnesses; $\varepsilon_{n}$, $\varepsilon_{s}$, and $\varepsilon_{t}$ represent the normal strain and two tangential strains, respectively.

The quadratic nominal stress criterion, which takes into account the combination of real and permitted stresses operating in various directions, can be defined by Eq. (20) and is used in the damage evolution phase of the simulation:

(20) $${\left(\frac{\left\langle t_{n}\right\rangle }{t_{n}^{0}}\right)}^{2}+{\left(\frac{t_{s}}{t_{s}^{0}}\right)}^{2}+{\left(\frac{t_{t}}{t_{t}^{0}}\right)}^{2}=1$$

where $\left\langle t_{n}\right\rangle$ is the secondary nominal stress; $t_{n}^{0}$, $t_{s}^{0}$, and $t_{t}^{0}$ denote the normal strength and two tangential strengths, respectively.

**Fig. 15:**
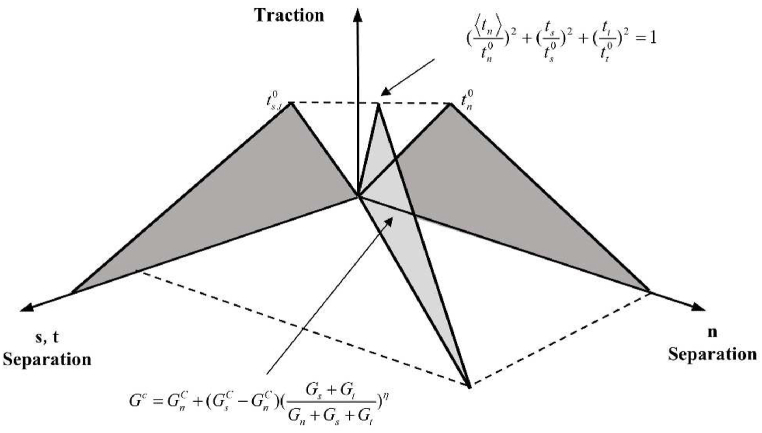
Cohesive unit damage model.

The cohesive unit will experience damage growth after it has reached the damage initiation point in accordance with the established law of damage evolution, and the damage evolution phase abides by the Benzeggagh-Kenane (B–K) fracture criterion [45], which is specified by Eq. (21):

(21) $$G^{c}=G_{n}^{C}+\left(G_{s}^{C}-G_{n}^{C}\right){\left(\frac{G_{s}+G_{t}}{G_{n}+G_{s}+G_{t}}\right)}^{\eta }$$

where $G_{n}^{c}$, $G_{s}^{c}$, and $G_{t}^{c}$ are the critical fracture energies required to initiate delamination damage in the normal direction and the two shear directions, respectively, and η is the coefficient of viscosity. The model used in the present research to describe cohesive unit damage in the hybrid model is shown in Fig. 15.

With reference to the interlayer performance parameters of comparable materials provided in the pertinent literature [46], the material parameters in the interlayer sub-model employed in this work are summarized in Table 5.

**Table 5:** Material parameters of the interlayer damage sub-model.

Characterizations	$t_{n}^{0}$ (Mpa)	$t_{s}^{0}$ (Mpa)	$t_{t}^{0}$ (Mpa)	$G_{n}^{c}$ (J/m^2^)	$G_{s}^{c}$ (J/m^2^)	$G_{t}^{c}$ (J/m^2^)	η
Value	54	70	70	504	1556	1556	2.284

### 3.3 Validation of model validity

**Fig. 16:**
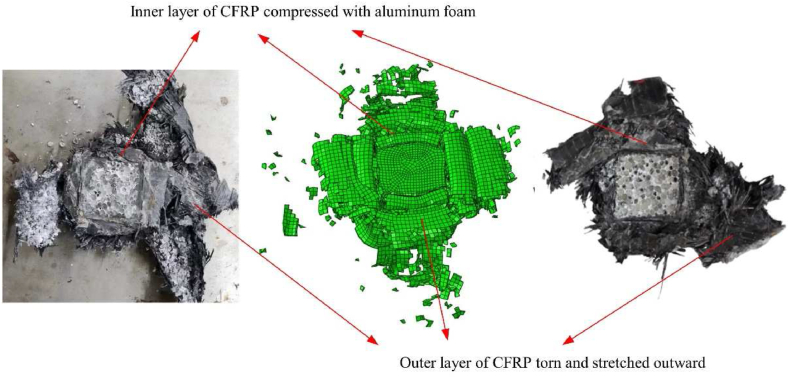
Comparison of axial crush test and simulation results.

The deformation diagrams from the simulation computation were compared with the outcomes from the test using the CFRP-FA-FW structure's finite element model created in the ABAQUS/Explicit program (Fig. 16): it can be stated that the CFRP-FA-FW failure modes in simulation and test are essentially the same.

**Fig. 17:**
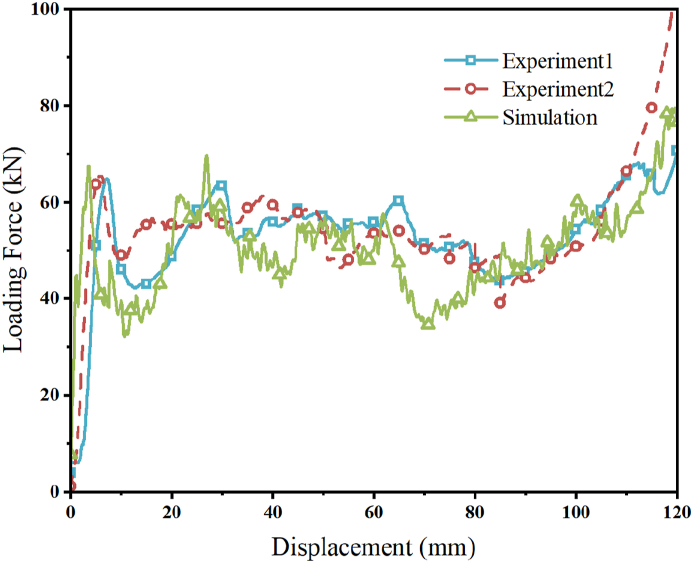
Comparison of test and simulation load-displacement curves.

Fig. 17 illustrates the load-displacement curves from the repeatability test and simulation. The curves from the test and simulation are largely in agreement, demonstrating that the simulation model is capable of simulating the failure deformation mode and mechanical response of the CFRP-FA-FW structure. The effectiveness of the created finite element model is demonstrated by way of the comparison (Table 6) of evaluation indices of energy absorption characteristics between the two repeating tests and the simulation, all of which fall within the permitted error range.

**Table 6:** Comparison of results between experiment and simulation.

Type	*EA*(kJ)	*PCF*(kN)	*MCF*(kN)
Experiment1	5.36	64.90	51.07
Experiment 2	5.42	65.40	51.64
Simulation	5.07	69.66	48.31
Error1	5.41 %	7.33 %	5.40 %
Error2	6.46 %	6.51 %	6.45 %

## 4 Energy absorption characteristics of the CFRP-FA-FW structure

### 4.1 Effect of the relative density of aluminum foam

A carbon fiber lay-up setup with a carbon fiber lay-up angle of [45°]_ns_ and a lay-up thickness of 2.5 mm was chosen to investigate the impact of the relative density of aluminum foam on the crashworthiness of the CFRP-FA-FW, and the finite element models of the CFRP-FA-FW with relative densities of aluminum foam of 0.25 g/cm^3^, 0.35 g/cm^3^, and 0.4 g/cm^3^ were established, respectively, to perform simulation computational analysis.

**Fig. 18:**
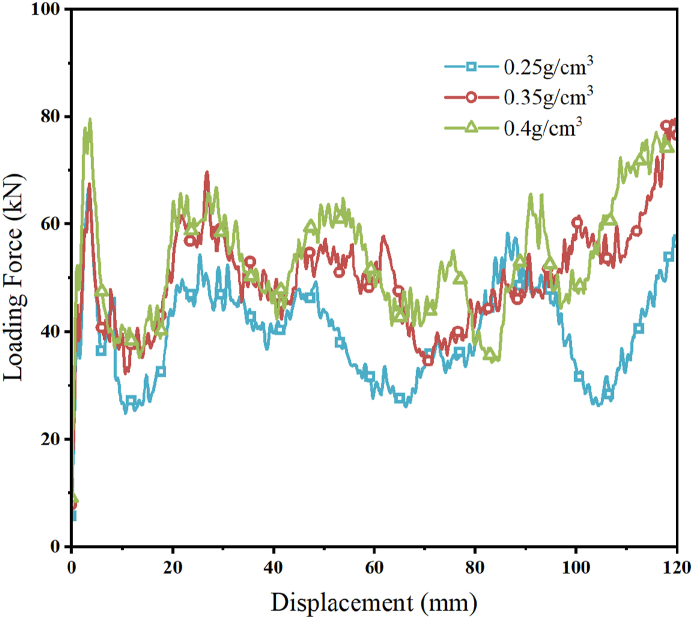
Load-displacement curves for different aluminum foam densities.

The load-displacement curves of CFRP-FA-FW constructions with various foam aluminum densities are shown in Fig. 18. The internal carbon fiber lay-up results in a tighter extrusion in the case of a compression collapse, which also causes load fluctuations in the load-displacement curves, thanks to the resin connection between the carbon fibers and the aluminum foam. The initial *PCF* of the CFRP-FA-FW under axial load has increased (Fig. 18), and the total energy absorption of the structure has also improved with an increase in the relative density of the aluminum foam.

The energy absorption indices for various densities of aluminum foam are listed in Table 7. The *PCFs* of the structures with relative aluminum foam densities of 0.35 g/cm^3^ and 0.4 g/cm^3^ are increased by 4.30 % and 19.04 %, respectively, compared to the structure with a relative density of 0.25 g/cm^3^, demonstrating that the greater the relative density of aluminum foam for the composite structure, the more significant the enhancement of the *PCF* is. In the meantime, as the relative density of aluminum foam increases, so does the amount of energy that CFRP-FA-FW is able to absorb. However, the improvement in the specific energy absorption of the CFRP-FA-FW is not significantly affected by the increased density of aluminum foam.

**Table 7:** Evaluation indices of energy-absorbing properties for different aluminum foam densities.

Densities (g/cm^3^)	*m* (g)	*EA* (kJ)	*SEA* (kJ/kg)	*MCF* (kN)	*PCF* (kN)	*CFE* (%)	*ULC*
0.25	194.04	4.18	21.54	39.81	66.79	59.60	0.18
0.35	217.64	5.07	23.31	48.31	69.66	69.35	0.13
0.4	229.45	5.35	23.33	50.99	79.51	64.13	0.14

### 4.2 Effect of CFRP lay-up angle

**Fig. 19:**
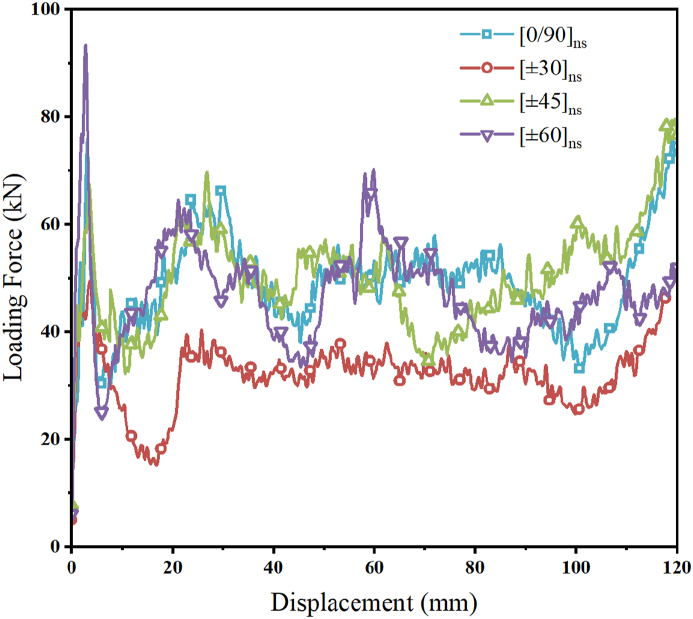
Load-displacement curves for different CFRP lay-up angles.

For the finite element simulation analysis, four typical carbon fiber lay-up angles ([0°/90°]_ns_, [±30°]_ns_, [±45°]_ns_, and [±60°]_ns_) are chosen. Fig. 19 displays the load-displacement curves for various lay-up angles, and it is clear that the [±60°]_ns_ lay-up angle has the largest initial *PCF* of the structure. This is primarily because as the lay-up angle increases, the strength of the carbon fibers in the direction perpendicular to the loading direction increases. The energy absorption and initial peak force for the CFRP-FA-FW structure with symmetric lay-up configuration are improved with an increase in lay-up angle.

The energy absorption quality evaluation indices for various CFRP lay-up angles are shown in Table 8. The structure with the lay-up angle of [±60°]_ns_ has the highest peak force at the start of loading; the structures with the lay-up angles of [0°/90°]_ns_ and [±45°]_ns_ present the highest energy absorption capacity; and the specific energy absorption (*SEA*) of these two structures increased by 53.54 % and 54.27 %, respectively, compared to that of the structure with the lay-up angle of [±30°]_ns_, indicating that the lay-up angles of [0°/90°]_ns_ and [±45°]_ns_ can endow the CFRP-FA-FW with a higher energy absorption capacity. The energy absorption process with a lay-up angle of [±60°]_ns_ causes significant variations, however, the energy dissipation process of the remaining three structures is smoother, according to the results of the combination of the *CFE* and undulation of load-carrying capacity *ULC*.

**Table 8:** Evaluation indices of energy absorption properties with different lay-up angles.

Layup angles	*m* (g)	*EA* (kJ)	*SEA* (kJ/kg)	*MCF* (kN)	*PCF* (kN)	*CFE* (%)	*ULC*
[0/90°]_ns_	217.64	5.05	23.20	48.05	75.81	63.38	0.13
[±30°]_ns_	217.64	3.29	15.11	31.33	49.50	63.29	0.13
[±45°]_ns_	217.64	5.07	23.31	48.31	69.66	69.35	0.13
[±60°]_ns_	217.64	4.92	22.62	46.88	93.30	50.25	0.16

### 4.3 Effect of CFRP thickness

For the simulation analysis, four alternative CFRP thicknesses are chosen, with the aluminum foam density of 0.35 g/cm^3^, the CFRP lay-up angle of [±45°]_ns_, and the CFRP thicknesses of 1.5 mm, 2 mm, 2.5 mm, and 3 mm, respectively.

**Fig. 20:**
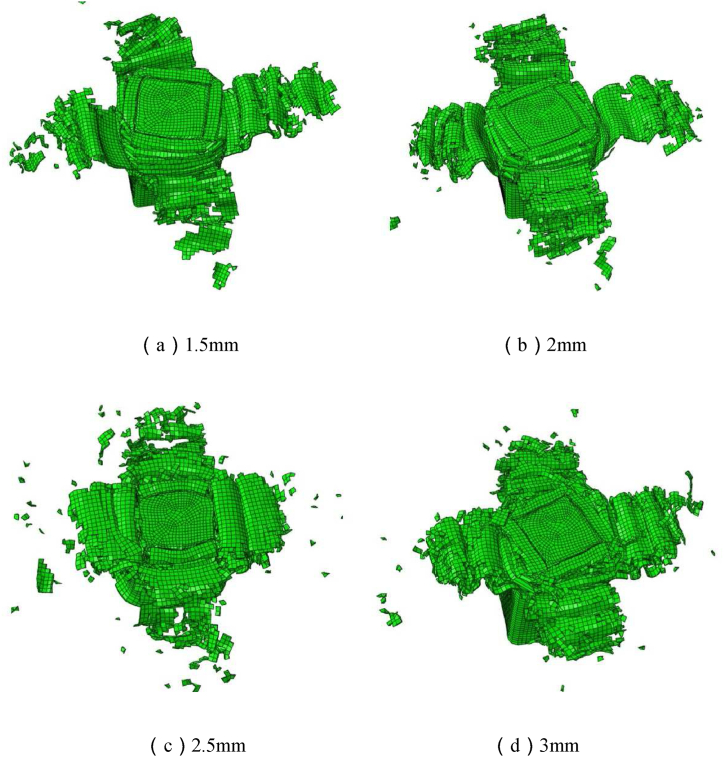
Failure diagrams of composite structures with different CFRP thicknesses.

**Fig. 21:**
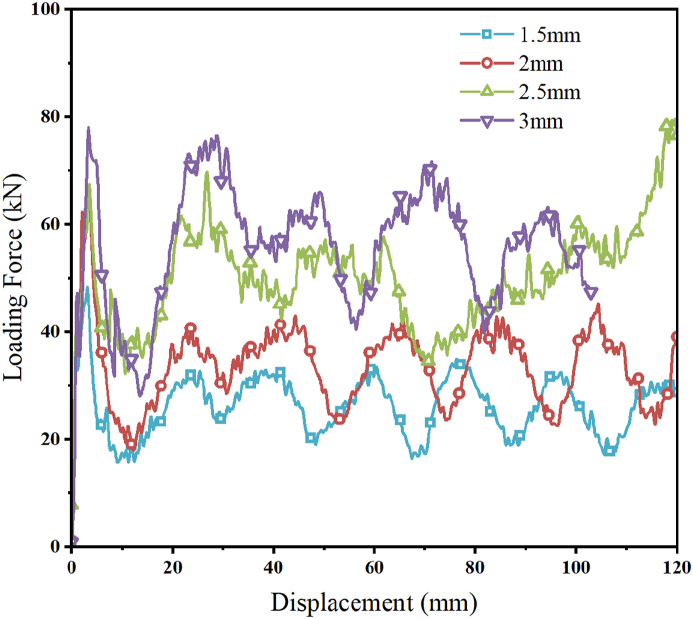
Load-displacement curves for different CFRP thicknesses.

The failure deformation diagrams of composite structures with four different CFRP thicknesses under axial load obtained by finite element simulation analysis are shown in Fig. 20(a–d), from which it can be seen that the change of carbon fiber thickness has less influence on the failure deformation pattern of CFRP-FA-FW under axial load, and the composite structures with four different CFRP thicknesses all show the tear and bloom failure deformation pattern, and the outer carbon fibers produce the delamination under the action of axial load. The load-displacement curves of the composite structure with four different CFRP thicknesses are shown in Fig. 21; as the thickness of the CFRP increases, so do the initial *PCF* and the amount of energy that the CFRP-FA-FW can absorb under axial load.

The comparison of the assessment indices of the energy absorption properties with various CFRP thicknesses is possible from the data listed in Table 9. The structure with the thickest CFRP (3 mm) has the biggest initial *PCF*, which is 29.68 kN. The axial loading capacity of the CFRP-FA-FW also rises as the thickness of the CFRP is increased. The specific energy absorption of the structures with CFRP thicknesses of 2.5 mm and 3 mm is greater than that of the structure with thickness of 1.5 mm by 36.80 % and 37.21 %, respectively. As can be observed, increasing the thickness of CFRP can significantly increase the ability of the CFRP-FA-FW to absorb energy. Combining the *CFE* and *ULC* data, the CFRP-FA-FW structure with a 2.5 mm thickness has the smoothest energy dissipation process among the four thicknesses.

**Table 9:** Evaluation indices of energy absorption properties with different CFRP thicknesses.

Layup thicknesses (mm)	*m* (g)	*EA* (kJ)	*SEA* (kJ/kg)	*MCF* (kN)	*PCF* (kN)	*CFE* (%)	*ULC*
1.5	162.51	2.77	17.04	26.37	48.34	54.55	0.17
2	189.89	3.55	18.68	33.78	67.32	50.17	0.18
2.5	217.64	5.07	23.31	48.31	69.66	69.35	0.13
3	245.75	5.75	23.38	54.73	78.02	70.14	0.15

## 5 Conclusion

The five structures, aluminum alloy square tube (Al–S), CFRP square tube (CFRP-S), aluminum foam-filled CFRP square tube (CFRP-FA), aluminum foam-filled aluminum tube (Al-FA), and T300-grade CFRP-FA-FW, were firstly compared through quasi-static compression tests. The results show that the inclusion of the foam-aluminum core during the curing and formation of the CFRP-FA-FW structure can result in a stronger bond between the carbon fibers and the aluminum foam. In addition, the crushing deformation of the carbon fiber itself, the plastic deformation of the aluminum foam, and the failure of the resin between the carbon fiber and the aluminum foam are all jointly involved in energy dissipation, which improves the energy efficiency. When compared to the simple composite structure CFRP-FA, the energy absorption and specific energy absorption of CFRP-FA-FW are both increased by 113.55 % and 60.73 %, respectively.

The CFRP-FA-FW structure, the relative density of aluminum foam, the CFRP lay-up angle, and the CFRP thickness were parametrically analyzed, revealing the following:

1) The change in the relative density of aluminum foam has a small effect on the failure deformation mode of the CFRP-FA-FW under axial loading, and the initial *PCF* of the CFRP-FA-FW is increased with the increase in relative density of aluminum foam. However, the increase in density of aluminum foam exerts no appreciable influence on the enhancement of the specific energy absorption of the CFRP-FA-FW;
2) The *PCF* of the CFRP-FA-FW increases with increasing lay-up angle for the symmetric lay-up setup. Higher energy absorption and a smoother energy absorption process were obtained at lay-up angles of [0°/90°]_ns_ and [±45°]_ns_;
3) As the thickness of the CFRP is increased, the initial *PCF* and energy absorption of the CFRP-FA-FW under axial loading both increases. This can increase the energy absorption capacity of the CFRP-FA-FW while also resulting in smoother energy absorption under impact.

CFRP-FA-FW is complex in terms of the manufacturing process, which requires precise control of the path, angle, and tension of the carbon fiber winding, as well as the curing conditions of the resin to ensure uniformity. These requirements require high investment in equipment, limiting its widespread use. However, with technological advances and cost reductions, the application of this material remains very promising. The theoretical and simulation models used in this paper to study CFRP-FA-FW are much simplified and the effect of surface cavities in the aluminum foam on the energy absorption characteristics of CFRP-FA-FW under axial loading was not considered. Numerical simulation methods will continue to be used and improved to predict the performance of CFRP-FA-FW more accurately in practical applications and to validate them in comparison with experimental results.

## CRediT authorship contribution statement

**Hui Zhou:** Writing – review & editing, Resources, Data curation, Conceptualization. **Yao Jiang:** Writing – original draft, Visualization, Validation, Conceptualization. **Guanghui Yang:** Writing – original draft, Visualization, Validation, Supervision, Formal analysis, Data curation, Conceptualization. **Suchao Xie:** Writing – review & editing, Validation, Supervision, Resources, Data curation, Conceptualization.

## Declaration of competing interest

The authors declare that they have no known competing financial interests or personal relationships that could have appeared to influence the work reported in this paper.
